# Medications, Deep Brain Stimulation, and Other Factors Influencing Impulse Control Disorders in Parkinson's Disease

**DOI:** 10.3389/fneur.2019.00086

**Published:** 2019-02-26

**Authors:** Robert S. Eisinger, Adolfo Ramirez-Zamora, Samuel Carbunaru, Brandon Ptak, Zhongxing Peng-Chen, Michael S. Okun, Aysegul Gunduz

**Affiliations:** ^1^Department of Neuroscience, University of Florida, Gainesville, FL, United States; ^2^Hospital Padre Hurtado, Facultad de Medicina, Clínica Alemana Universidad del Desarrollo, Santiago, Chile; ^3^Department of Neurology, Fixel Center for Neurological Diseases, University of Florida, Gainesville, FL, United States; ^4^Department of Biomedical Engineering, University of Florida, Gainesville, FL, United States

**Keywords:** impulse control disorder, Parkinson's disease, impulsivity, dopaminergic medications, deep brain stimulation

## Abstract

Impulse control disorders (ICDs) in Parkinson's disease (PD) have a high cumulative incidence and negatively impact quality of life. ICDs are influenced by a complex interaction of multiple factors. Although it is now well-recognized that dopaminergic treatments and especially dopamine agonists underpin many ICDs, medications alone are not the sole cause. Susceptibility to ICD is increased in the setting of PD. While causality can be challenging to ascertain, a wide range of modifiable and non-modifiable risk factors have been linked to ICDs. Common characteristics of PD patients with ICDs have been consistently identified across many studies; for example, males with an early age of PD onset and dopamine agonist use have a higher risk of ICD. However, not all cases of ICDs in PD can be directly attributable to dopamine, and studies have concluded that additional factors such as genetics, smoking, and/or depression may be more predictive. Beyond dopamine, other ICD associations have been described but remain difficult to explain, including deep brain stimulation surgery, especially in the setting of a reduction in dopaminergic medication use. In this review, we will summarize the demographic, genetic, behavioral, and clinical contributions potentially influencing ICD onset in PD. These associations may inspire future preventative or therapeutic strategies.

## Introduction

**Figure 1:**
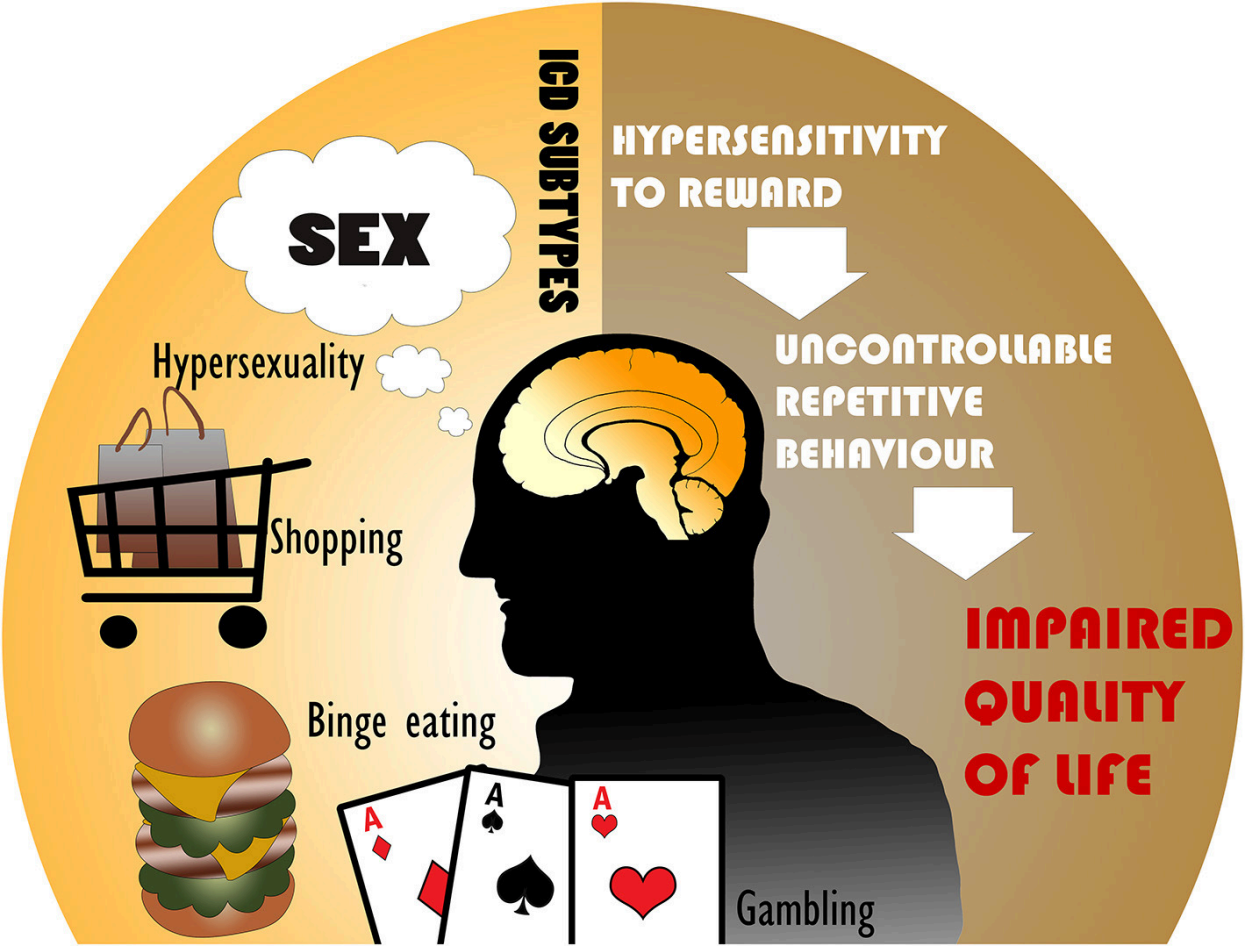
The most common impulse control disorders (ICDs) in Parkinson's disease include hypersexuality, compulsive shopping, binge eating, and pathological gambling. ICDs are associated with hypersensitivity to reward and uncontrollable repetitive behaviors, leading to an impaired quality of life.

Parkinson's disease (PD) is a neurodegenerative disorder of dopamine-producing neurons in the substantia nigra and also includes widespread dysfunction throughout motor and non-motor brain circuits (1). PD motor symptoms such as tremor, bradykinesia, and rigidity are well-recognized (2), however PD is strongly associated with several non-motor symptoms as well. In contrast to the motor symptoms, non-motor symptoms are understudied and encompass cognitive, autonomic, and neuropsychiatric abnormalities (3). Among these problems, PD patients may experience changes in affective or goal-directed behaviors that can manifest as impulsivity. Impulse control disorders (ICDs) are commonly characterized by four major subtypes: pathological gambling, hypersexuality, compulsive shopping, and binge eating, but can also include punding, hobbyism, and dopamine dysregulation syndrome (DDS), which may be separated into ICD-related behaviors (ICD-RB) in some classifications (Figure 1) (4, 5). These behaviors as a whole may also be referred to as impulsive-compulsive behaviors (ICBs), but in this paper we refer to all subtypes collectively as ICDs.

Those with ICDs have an inability to resist inappropriate internal drives, and these may result in repetitive behaviors with harmful consequences that can impact quality of life for both patients and caregivers (6). A recent, large multicenter study of ICDs found a 5-year cumulative incidence of 46.1% (7). It has been estimated that ICDs affect 13.6% of PD patients, although this number varies widely across samples (8). Nonetheless, the true prevalence may be higher, especially since PD patients tend to underreport embarrassing and in many cases pleasurable behaviors, (9) and may lack insight into their problematic behaviors (10). In one study, only one quarter of PD patients experiencing ICDs were clinically identified (11). Patients may also experience sub-clinical impulsivities (9, 12).

**Figure 2:**
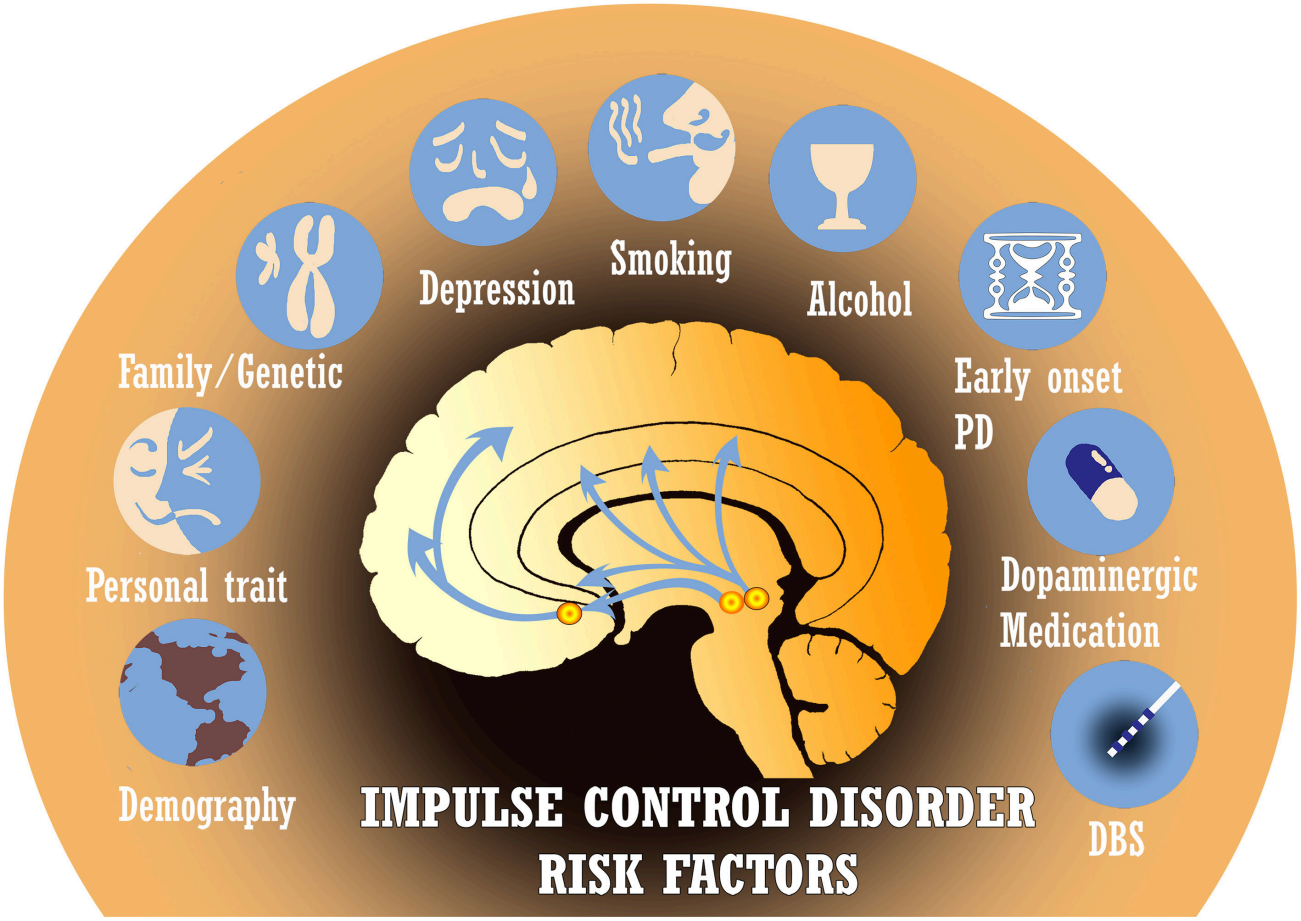
There are many established risk factors for impulse control disorders (ICDs) in Parkinson's disease (PD), including demographics, personality traits, genetic predisposition, depression, tobacco/alcohol use, age of disease onset, dopaminergic medications, and deep brain stimulation (DBS). Several other risk factors under investigation are not depicted.

ICDs in PD have classically been attributed to long-term exposure to dopaminergic medications such as levodopa and dopamine agonists. These drugs alter the pathophysiology of reward-based neural networks (13). However, other pertinent risk factors have been identified and include gender, country of residence, age of PD onset, disease duration, alcohol/tobacco use, family history of impulsivity, genetic factors, non-dopaminergic medications, deep brain stimulation, personality traits, and more (Figure 2) (5, 8). Several recent studies have even observed these non-dopaminergic factors as significantly contributing most to the variance in impulse control disorder risk. Recognizing the multiple associations that have been reported in the literature is crucial in order to identify areas for further investigation of the etiology and management of ICDs.

In this review, we provide a summary of the known ICD risk factors and associations with a focus on five main areas: demographics; medical and surgical associations; premorbidities and comorbidities; family history and genetics; and personality traits. We also include a brief section on neural correlates and cognitive changes associated with ICDs as observed through behavioral studies, human imaging, and electrophysiology. We conclude by highlighting that dopamine alone cannot account for all ICDs, and we point out limitations of present studies which may help to motivate future investigations.

## Demographics

### Gender

In general, proportionally more male than female PD patients screen positive for ICDs (5, 8, 14–20). One large PD study of 32 sites in Italy found that 223 (32.5%) of 686 males and 83 (21.7%) of 383 females screened ICD-positive (5). Such gender effects have been widely reported (21–23). For instance, the DOMINION study of 3090 PD patients found that males comprised 64% of both ICD+ and ICD- patients, although the prevalence of specific ICD subtypes differed by gender (8). It is difficult to determine if gender is decisively a risk factor for ICDs in PD, or if the higher prevalence in males with ICDs is largely observed due to the overall demographics of the PD population, which is predominantly male (24). Additionally, differences in the expression of ICD behaviors could contribute to under-reporting.

Gender differences can also arise when examining specific subtypes of ICDs. For instance, patients with compulsive sexual behavior are predominantly male (8, 25–28). On the other hand, patients with compulsive shopping and binge eating are predominantly female, indicating that biological and social factors may influence the expression of ICD behaviors (8, 25–27). These gender patterns for compulsive sexual behavior and binge eating also hold true in non-PD ICD populations (27). A limited number of studies suggest that pathological gambling occurs more in males with PD (29) and in the general population (27). Finally, although few studies have examined gender differences across PD patients with other ICBs, there seems to be a male predominance for punding and hobbyism (30–32) and a lack of gender difference for rates of DDS (33).

### Age, Age at Diagnosis, and Disease Duration

Most studies are in agreement that younger PD patients have an increased risk of ICDs (5, 8, 14–16, 19, 21, 23, 26, 31, 34–39). Patients with ICDs are also usually younger at PD onset and at the time of diagnosis (5, 14, 16, 21, 26). Therefore, early-onset PD and those with longer disease duration tend to have a higher risk of ICDs (5, 20, 39, 40). It is possible that those who have been diagnosed at younger ages and have longer disease duration consequentially have more exposure to dopaminergic medications, potentially increasing their risk for developing an ICD. However, despite robust associations between ICDs and dopaminergic medication use, other studies have failed to identify a relationship between ICDs and age or disease duration (8, 16, 21, 22, 27), and so the effect of dopamine treatment cannot not fully explain this association. To investigate such factors simultaneously, multivariate analysis must be used to measure independent effects across multiple variables. For example, a dearth of studies have collectively shown persistent age-dependent effects even when controlling for DA use (8). Interestingly, in non-PD populations, ICDs represent a category of diseases with a younger age of onset relative to other DSM-V disorders (41), further highlighting the independent effect of age on ICDs.

### Country of Residence

**Figure 3:**
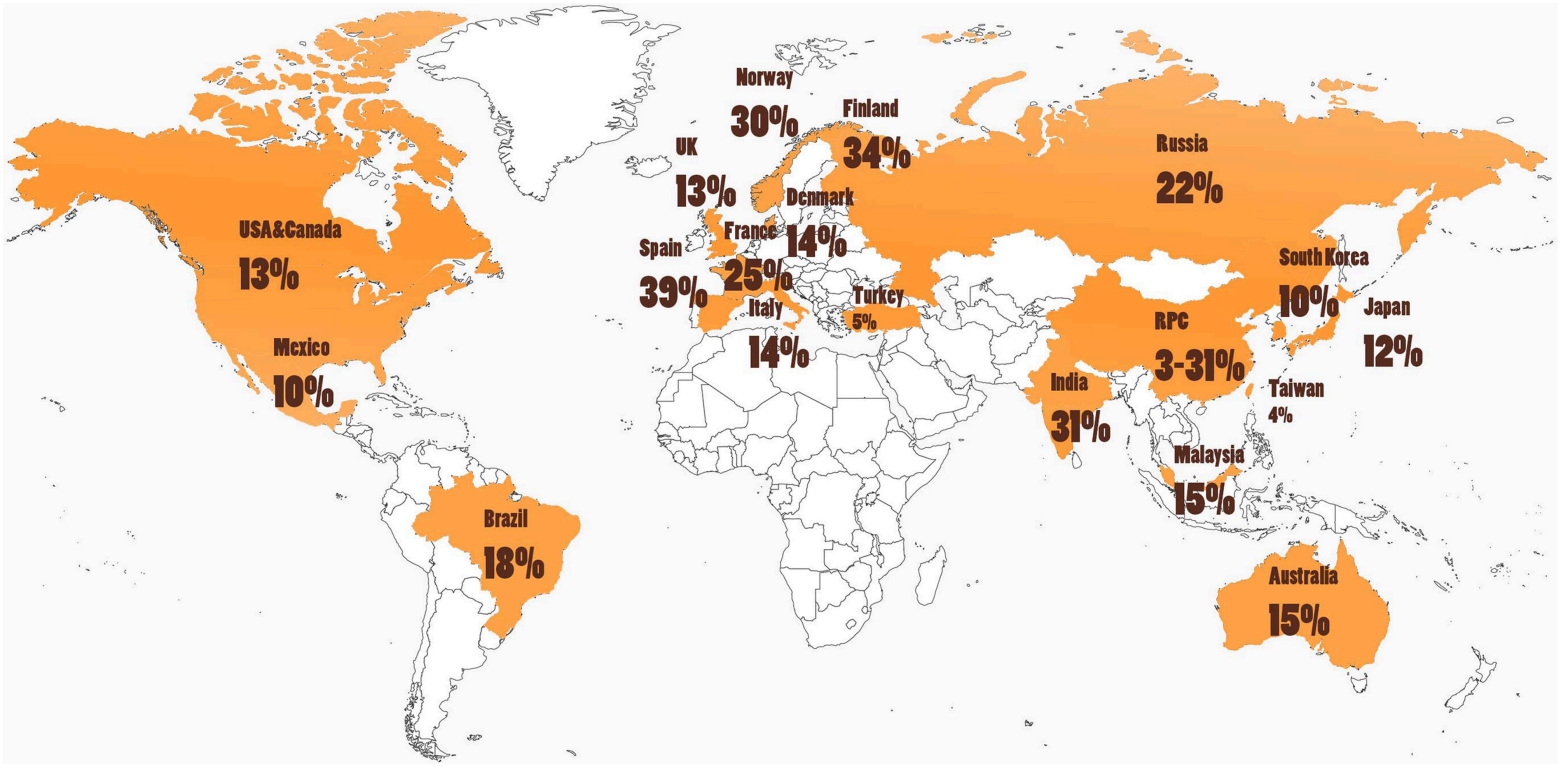
Cultural and environmental factors may influence ICD risk, as rates of impulse control disorders (ICDs) in people with Parkinson's disease (PD) around the world are highly variable (see Table 1 and text). Further studies are needed that investigate ICD rates in South America, Africa, and areas in Europe and Asia.

Cultural and other environmental differences may affect both the incidence and presentation of ICD behaviors (25, 42). When evaluating PD-associated ICDs across different regions in the world, ICD prevalence varies widely as seen in Table 1 and depicted in Figure 3. For example, in one large multicenter study, ICDs were more common in the United States (US) vs. Canadian PD populations, with pathological gambling, and compulsive buying reported more commonly in US patients (8). Asian countries such as China, Taiwan, and South Korea tend to show a lower prevalence of PD ICDs (22, 42, 43). However, India was noted to have a particularly high prevalence of ICDs at 31.6% (45). Interestingly, most of the European nations evaluated had a PD ICD prevalence greater than that of the US(5, 18, 35, 37, 49). A study of Finnish PD patients found a prevalence of pathological gambling seven times higher than in the general Finnish population (36). Central and South American nations have revealed a prevalence near equivalent or moderately higher than that of the US (15, 46). Interestingly, punding is the most common of the ICDs reported in Turkey (44).

**Table 1:** Prevalence rates of ICDs across the world.

**Country**	**Percentage of PD patients exhibiting ICD**
China	3.53% of 400 (22), 7.0% of 213 (19), 31% of 142 (38)
Taiwan	4.5% of 268 (42)
South Korea	10.1% of 1167 (43)
Japan	12.9% of 118 (23)
Malaysia	15.4% of 195 (20)
Turkey	5.9% of 554 (44)
India	31.6% of 305 (45)
United States & Canada^*^	13.6% of 3090 (8)
United States	6.6% of 272 (11), 12.8% of 250 (39)
Australia	15% of 100 (6)
Brazil	18.4% of 152 (15)
Mexico	10.6% of 300 (46)
UK	17.8 of 500 (47), 13.7% of 297 (48)
Russia	22.4% of 246 (49)
Finland	34.8% of 575 (36)
Norway	30.4% of 125 (50)
Denmark	14.9% of 490 (31)
Spain	39% of 233 (37)
Italy	28% of 1069 (5), 7.6% of 1063 (28), 8.1% of 805 (14),
France	25% of 203 (51)

* *Higher in the US. PD: Parkinson's disease; ICD: Impulse control disorder*.

Comparison across studies assessing the prevalence of ICDs has been severely limited by differences in study design, clinical criteria, and ICD screening tools. Self-report questionnaires may lead to a sampling bias (22). Despite such limitations, potential cultural, and geographic differences bring into question the role of environmental factors on ICDs. Several studies have noted such differences and attribute them to cultural factors generally without offering more specific ideas or explanations (52). One study of a sample of early-onset PD patients from Spain suggested that the use of technologies in younger populations contributed to higher rates of hobbyism, as this was also the highest impulse control behavior identified in a non-PD age-matched control cohort (25). In the US, casinos and shopping malls are more accessible than in Canada, which might explain the higher prevalence of gambling and compulsive buying. Yet, it is hard to draw conclusions on the directionality of this relationship, since the availability of casinos and shopping malls may be related to intrinsic cultural differences between the populations.

It is important to consider that most studies characterize ICD prevalence rates in PD patients without comparison to rates in a non-PD sample. It is also important to note that PD and ICD management strategies may differ throughout the world. For instance, the dopaminergic medication pramipexole has only been available in China since 2007 (22). Nonetheless, differences can be seen across many countries and ICD subtypes, but explanations that capture these differences are mostly speculative, and non-empirical. Using standardized methodologies, future work could be directed to study a region with a relatively low rate of ICDs and one with a relatively high rate of ICDs as a method to uncover potential preventative strategies.

## Medication and Surgical Associations

### Dopaminergic Medications

The association between dopaminergic medications and ICDs is the most documented of all associations. Many well-designed studies have collectively observed that higher dopamine use through either dopamine agonists or levodopa increases the risk of developing ICDs (5, 8, 14, 15, 21, 22, 25, 26, 34). In the DOMINION study, dopamine treatment was the risk factor with the highest odds ratio for ICD risk in a multivariable analysis with a value of 2.72 (8). When extending the analysis to ICD subtypes the odds ranged from 2.15 (pathological gambling) to 3.34 (binge eating). Dopamine treatment was associated with a seven-fold increased risk of ICBs in one study of early-onset PD patients (25). Other dopaminergic medications, such as monoamine oxidase-B inhibitors (MAOB-Is), have not demonstrated such clear results. While some studies have found evidence of an association between MAOB-Is and ICDs (37), others have reported no relationship (15, 25). A few case studies have shown MAOB-I-induced hypersexuality and pathological gambling (53, 54). The role of MAOBIs in ICDs is attributed to its effect on behavioral plasticity and personality traits such as impulsivity and aggression (55).

The physiological connection between dopaminergic medications and ICDs has been published extensively (56, 57). Briefly, dopamine differentially modulates impulsivity and behavioral addictions, likely through its involvement in neural pathways of reward and punishment (13). Many of the commonly prescribed dopamine agonists such as pramipexole and ropinirole have a higher affinity for D3 than D1/D2 receptors, leading to significant binding outside of the targeted nigrostriatal projections (57). The association between ICDs and dopaminergic drugs suggests an overactivation of the mesolimbic dopaminergic system that underlies pathological responses to natural rewards. Dopamine replacement therapies restore normal dopamine levels in motor pathways but may adversely stimulate the relatively preserved mesocorticolimbic system, particularly in genetically-predisposed or otherwise-vulnerable patients. This may result in patients experiencing hypersensitivity to rewards. Additionally, there has been compelling evidence suggesting that other brain structures and neurotransmitters may be critical to the development of these disorders in PD (58–60).

Despite the vast body of evidence supporting the neurobiological plausibility of dopaminergic overdosing of non-motor pathways and subsequent behavioral abnormalities, a direct causality has been challenged by numerous studies that hint at more complex dopamine-ICD relationships. First, several studies have not found the simple association between impulsivity or ICDs and dopamine agonist or levodopa use (6, 18, 23, 38, 61). Secondly, there may be differential effects across the various dopaminergic medications and their routes of delivery. For instance, some studies find a graded relationship between ICDs and levodopa dose but not between ICDs and dopamine agonist dose (8, 23, 62). Others find the opposite, concluding a graded relationship between dopamine agonists and ICDs but not between levodopa and ICDs (25, 35). Still other studies show that dopamine-ICD associations are statistically present only when considering a combination of dopamine agonists and levodopa using a total levodopa equivalent daily dose (LEDD), but not with a dopamine agonist LEDD alone (27, 31). Numerous reports have shown differences in oral vs. transdermal or short-acting vs. long-acting routes of dopaminergic medication delivery (37, 63–65), suggesting some importance for pharmacokinetics of non-continuous vs. continuous dopamine receptor stimulation (45). Third, individuals with restless leg syndrome (RLS) treated with dopaminergic agonists show lower rates of ICDs than PD patients (35), implying that in PD patients certain susceptibility factors are likely at play. For instance, a history of ICDs prior to PD diagnosis is a contributing risk factor for the development of ICDs after dopamine agonist use for PD treatment (11). Fourth, withdrawal or reduction of dopaminergic agents after ICD onset does not always predictably reverse an ICD (66, 67), suggesting some persistent dopaminergic effect [e.g., PD patients with pathological gambling still show elevated presynaptic ventral striatal dopamine release off-medication (68)] or that ICD pathophysiology critically implicates factors beyond dopamine. In one large study, more than half of ICDs persisted even 1 year after discontinuation of dopamine agonists (7). This situation can be compared to the fact that in multiple regression models, dopaminergic medications do not explain the bulk of variability in impulsiveness. For example, a Danish model including sex, age, age at PD onset, motor symptomology, total dopaminergic medication use, dopamine agonist use, smoking, depression, and personality traits only explained at most 31.2% of ICD variance (31). Fifth, many studies have demonstrated equivalent risk for dopaminergic medication use and the various ICD subtypes (40, 63, 69, 70), solidifying the necessity of susceptibility or other factors that, for instance, predispose to pathological gambling vs. hypersexuality. Sixth, there is a lack of evidence that dopaminergic blockade improves impulsive behaviors (64). In fact, in one account aripiprazole worsened pathological gambling in a PD patient (71). Finally, not all patients using dopaminergic medications report ICDs (72), and conversely, ICDs have been reported in PD patients prior to starting dopaminergic treatments (7, 52, 73). These are all important observations that motivate exploration of ICD associations beyond dopaminergic medications.

Although considering the impact of dopaminergic treatment on impulsive behaviors in PD is supported by many large studies, other studies have concluded that there are greater roles for non-dopaminergic factors. For example, a study of PD patients found that smoking was a stronger predictor for the presence of ICD than was dopamine agonist use, with smoking leading to a three-fold increase in the risk for ICD (31). In another study of 575 patients, depression played a larger role than sex, age, age of disease onset, alcohol use, or medication in explaining the variance in ICDs (36). These types of analyses are only permitted through multiple variable models, which are commonly missing in numerous papers of ICD associations as a result of low sample size (23, 42, 66, 74). Results from multiple variable models may significantly differ from those in univariate models (15, 17). For instance, younger patients tend to use dopamine agonists more and so these variables may not be independent contributors to ICD risk.

Moving forward, ascertaining the role of dopaminergic agents in ICD-onset requires more robust investigation. To develop a model of causality classically requires satisfying several criteria, especially association, time order, and biological gradient (75). While the association between dopamine and ICDs has been realized extensively, rigorous statistical approaches controlling for other associated interrelated factors should be used. Although difficult to tease apart, the temporal sequence of dopamine use and ICDs must also be clearly established using longitudinal, prospective studies. Currently, whether or not a biological gradient truly exists—that is, whether dopaminergic doses independently contribute to ICD onset—remains an open question for future investigation. Controlled studies are therefore needed, particularly because the link between dopamine agonists and ICDs has been more firmly established. Hence, PD individuals at higher risk of ICDs included in recent studies may not have been prescribed dopamine agonists, leading to an important selection bias (5, 36). Similarly, controlled studies are needed because heterogeneity in ICD subtypes is not negligible. For example, dopamine agonists may be more associated with specific subtypes in select PD populations (43). Future work could address these current shortcomings and considerations.

#### Nondopaminergic Medications

Non-motor symptoms in PD involve more than just dopamine (76), but the influence of non-dopaminergic medications specifically on ICDs is unclear. A large percentage of studies that evaluate ICDs are retrospective cohort studies and since some non-dopaminergic medications are used as treatments for ICDs (e.g., antidepressants and antipsychotics) it is difficult to determine the directionality of reported associations. Long-term treatment with some of these medications, such as antidepressants, has been associated with overactivity of dopaminergic neuropathways (77). One study found that after accounting for possible confounding variables including motor score, age, gender, and disease duration, antidepressants were significantly associated with total impulsivity score, and sleep inducers were significantly associated with a binge eating impulsivity subscore (78). Few case reports have reported non-dopaminergic medications inducing ICDs in PD (79). Other studies have found no association between ICDs and commonly used non-dopaminergic medications such as benzodiazapines and antidepressants (14, 21, 22). The results are variable and a specific drug effect is difficult to determine as patients may be using different combinations of these drugs. Similarly, studies have suggested that GABAergic neurotransmission is associated with impulsivity, which is the target of common medications such as benzodiazepines (80). Given the scarcity of studies, it is hard to conclude if non-dopaminergic medications have any major association with ICDs; however, it is important to recognize the overlap of medication targets with brain pathways important to impulsivity.

### Deep Brain Stimulation (DBS)

The relationship between DBS and ICDs is complex with conflicting reports. The mechanisms behind the motor and non-motor effects of subthalamic nucleus (STN) DBS are under investigation and remain of great interest, especially since they can reveal further insight into functional networks including those involved in impulsivity, reward, and inhibition (81). With regards to ICDs after DBS, studies have found contrasting results ranging from observable benefit, worsening, or no change (82, 83). STN DBS may improve ICDs indirectly because of marked reductions in dopaminergic medication from the positive effect of DBS on reducing motor symptoms (84–89). For instance, one large, longitudinal prospective study of 110 PD patients showed a decrease in DDS behaviors 1 year after STN-DBS (90). Another large, longitudinal study found a significant decrease in rates of hypersexuality, pathological gambling, and DDS after STN-DBS, with ICDs remitting in 69% of patients but persisting in 31% (91).

Nonetheless, binge eating, impulsive aggressive behavior, pathological gambling, hypersexuality, and dopaminergic medication addictions after STN stimulation have been previously reported (92–98), and 67% of Parkinson Study Group (PSG) centers reported the occurrence of *de novo* ICDs after DBS surgery, despite only 13% utilizing consistent and formal ICD assessment tools (99). Animal work and preclinical models tend to corroborate and support the possibility of increased impulsivity after STN lesions (100). One study demonstrated postoperative persistence or worsening in 71% of patients with preoperative ICDs (101), and a systematic review found that across a total of 19 studies, the mean prevalence of new ICDs after DBS was around 15% (102). *De novo* ICDs after surgery may be associated with specific independent risk factors such as younger age, lower dyskinesia improvement, and schizoid personality traits (91). Long-term follow-up is mostly lacking, but one small study found groups of patients with new ICD-onset shortly after STN-DBS as well as several years after surgery (103). In other cases, worsening of impulsivity symptoms occurred after surgery but with eventual resolution, such as in one study of pathological gambling and STN DBS (85). The globus pallidus internus (GPi) is becoming another popular anatomical target for PD DBS, and although there are fewer DBS studies of the GPi in general, it should be noted that there are also reports of new-onset ICDs after GPi DBS (82, 104, 105).

It remains unclear why STN stimulation can affect ICDs, but it may be related to decision-making impairment and adverse influences on the reward processing function of the STN, particularly in situations of high conflict [for review, see Eisinger et al. (81)]. In this manner, the STN regulates behavior by providing a stopping mechanism within the cortico-striato-thalamo-cortical circuit (106). Beyond basic motor control, the STN is notably involved in numerous non-motor functions and lesions impact decision-making and inhibition (107–110). Both motor impulsivity and impulsive decision-making can contribute to ICDs (106, 107, 111). Ultimately, ICDs are complex and relate to elements beyond impulsivity including novelty seeking, depression, anxiety, and the many other factors discussed in this paper; thus, isolating the effect of stimulation can be difficult. For example, one study reported that a patient repeatedly experienced “morphine-like” effects while switching between off and on STN DBS (112), and cases of suicide have also been reported after DBS (96), some of which are thought to be directly related to impulsivity (113, 114). Another interesting study reported a case of trichotillomania that was right-dominant preoperatively but left-dominant postoperatively (115). Postoperative behavioral changes can be widespread and complex, and therefore the underlying pathophysiology of ICDs in the setting of DBS is wide open to continued investigation.

Interestingly, several reports have described a higher frequency of impulsive behaviors in DBS patients despite a reduction of dopaminergic medications (116, 117). In the setting of increasing dopaminergic medications and DBS together, it may be difficult to determine which factor, if any, more so accounts for new-onset ICD (93). In one large study, a prior history of DBS did not seem to confer an additional risk for ICD overall (8). Yet this may differ with specific ICD subtypes, as one paper, for instance, found that DBS—but not dopamine use—predicted postoperative binge eating (92). Nonetheless, other authors have concluded that dopamine agonist use and DBS carry a similar risk for ICD (116, 117). If dopaminergic-induced ICDs are related to dysfunction of reward pathways, it is possible that stimulation-induced ICDs have a similar underlying mechanism (102). In addition, research shows that STN DBS impairs impulse suppression when patients are either on or off dopaminergic medications (118, 119). Not all STN surgeries are comparable, as lead position and active contact configurations may vary considerably across subjects (120–122). This may in part account for the unpredictable effect of DBS on ICDs, and further studies are warranted.

## Premorbidities and Comorbidities

### Alcohol and Smoking

Similar to other risk factors, studies of the effect of alcohol on ICDs have presented mixed results. While some studies have found that PD patients with ICDs are more likely to regularly consume alcohol (22), the DOMINION study and others found no such difference (5, 8, 27, 38). Another study found no difference in alcohol consumption between early-onset PD patients with or without ICDs (25). The effect of alcohol has also been examined for specific subtypes of ICDs. In non-PD populations, a large study by the National Epidemiologic Survey on Alcohol and Related Conditions stated that around 73% percent of pathological gamblers have an alcohol use disorder (123). This relationship holds true for PD populations as well, with PD pathological gambling patients being 6.9 times more likely to have a personal or immediate family history of alcohol use disorders (124). The trend for smoking as a risk factor for ICDs seems to be more consistent, showing that PD patients with ICDs are more likely to be current, regular, or past smokers (5, 8, 15, 27, 31, 36, 38). Few studies have found no effect of cigarette use (22). Although the reason for this association is not clear, it has been hypothesized that it could be related to a decrease in both D2 receptors and dopaminergic cell activity similar to what is observed in patients with addictions (15).

### Family History and Genetics

It has been shown that patients with a family history of impulsivity are at greater risk of developing addictions (41). It is difficult to determine if this is due to genetic factors that affect impulsivity-related neural pathways, or because of the home environment. Family history has been commonly regarded as a risk factor for ICDs in PD populations, yet only a few studies have been conducted on this issue. The largest study to date was Weintraub et al. which observed that PD patients with a family history of gambling and alcohol use have higher rates of ICDs (8). The odds ratio for having a family history of gambling was considerably high (2.08), scoring above levodopa treatment (1.51) and smoking (1.70) (8). Another study that investigated a sub-population of PD patients with restless leg syndrome also found that a family history of gambling was associated with developing an ICD (125). Although the association between ICDs and family history has been examined, additional studies are needed to draw parallels to PD populations. Understanding a patient's family history might offer a more clear picture of susceptibility and thus likelihood to develop an ICD.

Genetics has also been proposed as a risk factor for ICDs. Several non-PD twin and adoption studies predicted the hereditability of pathological gambling and substance abuse to be around 60% (126, 127). A large longitudinal cohort of *de novo* PD patients obtained a similar value of 57% (128). In recent years, polymorphisms in dopamine receptors (DR) have been studied as possible explanations for ICDs. DRD1 and DRD2 are both associated with the motor effects of dopamine, DRD3 with behavioral effects and addictions, and DRD4 and DRD5 with attention deficit disorders (129). A common DR polymorphism studied is the DRD2 Taq1a, which substitutes glutamic acid for lysine in a serine/threonine kinase, possibly decreasing substrate binding in the DRD2 receptor (130), however some studies have not found this association (129–132). Other polymorphisms associated with ICDs include: DRD1 rs4867798, DRD1 rs4532, GRIN2B rs7301328, DRD3 p.S9G, and HTR2Ac.102T > C (129, 132, 133). Recently, a study by Kraemmer et al. suggested expanding the investigation of PD polymorphisms in DR genes to also include other genes such as DDC, which has also been linked to impulsivity (128). Parkin-associated PD patients also appear to be at a higher risk specifically for compulsive shopping, binge eating, and punding/hobbyism (134). Overall more gene-environment studies are needed to reach more firm conclusions and ideally develop models to identify at-risk patients.

### Personality Traits

Not surprisingly, impulsivity is the most commonly-studied personality trait in PD patients with ICDs. In this manner, impulsivity is defined as “actions that are poorly conceived, prematurely expressed, unduly risky, or inappropriate to the situation and that often result in undesirable outcomes” (135) and can be assessed using questionnaires or behavioral paradigms. Many studies have found a positive association with impulsivity and ICDs (27, 136). Levels of impulsivity are related to severity of impulse control disorders (137). Similarly, novelty seeking has also been discussed in previous reports given its interrelatedness to impulsivity (16, 27) and its emergence after dopamine therapies (138). As expected, PD patients with ICDs are more likely to choose novel options and are more attracted to novel stimuli compared to PD patients without ICDs (16). Poor social behavior and obsessive-compulsive features have also been linked to ICDs (27), although the results have been mixed (42).

The greatest differences in personality traits and ICDs arise when studying specific subtypes of ICDs. A literature review in non-PD patients that evaluated seven empirically-validated studies on pathological gambling found that coping styles, impulsivity, sensation seeking, and engaging in maladaptive delinquent/illegal activities are all risk factors for pathological gambling (139). Similar results have been observed in PD populations, in which pathological gambling has been linked to bizarre ideation, cynicism, and a tendency to lie (124, 140). A small case series identified a preliminary association between hypersexuality and delusional jealousy (28). Patients with ICDs also tend to show higher neuroticism, lower agreeableness, less conscientiousness, more paranoid ideation, and more negative emotionality, as well as more borderline, schizoid, and/or schizotypal traits (21, 31, 91, 141). Some studies have drawn parallels between the personalities of PD patients with ICDs and individuals with substance abuse (141). Future work should explore independent contributions of genetics and personality traits for the development of ICDs.

### Comorbidities and Other Clinical Associations

PD patients with ICDs have reduced quality of life and are more likely to exhibit prior or ongoing anxiety and depression (5, 6, 17, 19, 27, 34, 36, 47). The same is true for ICD patients with early onset PD (25). The directionality of the association is unclear, since it is often difficult to predict whether these comorbidities are a risk factor for the development of ICDs or results from ICD behavior (18, 25). Although a general link between PD and depression has been established, the interpretation of these results is complicated by the fact that rates of depression are similar between drug-naïve PD patients and non-PD individuals (18, 52, 142). Nonetheless, as mentioned above, depression levels may explain ICDs more so than other common associations such as dopaminergic medications (36).

Sleep disorders have also been investigated, with some studies finding more sleep impairment and daytime sleepiness in PD patients with ICDs (5, 65). Although some studies that defined sleep disorders through questionnaires found an association with ICDs, more recent studies—including those that have screened sleep disorders through polysomnography exams—have revealed inconsistent results (15, 78, 143, 144). Patients with ICDs may also have more restless leg syndrome (65). The association between sleep disorders and ICDs continues to be debated and thus larger, prospective studies are needed to clarify this relationship.

Other comorbidities that have been evaluated include diabetes mellitus, hypertension, coronary heart disease, and constipation, yet no consistent associations with PD ICD have been found (15). Whether or not PD patients with ICDs exhibit greater motor symptom severity is also controversial with reports of positive, negative, and null results (5, 15, 19, 27, 31, 145). One study found specifically that freezing of gait is associated with higher rates of ICD (38), although another study examined motor subtypes and found no significant difference in ICD rates between postural instability and gait disorder dominant (PIGD) and non-PIGD PD patients (47). Other, less common and less consistent associations have been described, including autonomic function (73), sexual function (5), apathy (5, 146), motivation (27), delusions (14), dementia (14), hallucinations (21), and illusions (21)—contrasting other studies that did not find such associations with hallucination (19, 23) or apathy (23), for example. However, these associations are important to recognize, as they may directly impact prevalence rates. For instance, some studies specifically exclude PD patients with dementia (21), which could thus lead to a higher ICD prevalence because patients with dementia tend to have lower rates of ICD. Therefore, risk factors for ICDs in PD may differ across studies depending on inclusion and exclusion criteria.

## Neural Substrate

### Imaging and Electrophysiological Alterations

Numerous imaging studies have been conducted with non-PD populations, however fewer studies have examined PD populations. Patients with PD in general and ICDs in particular have prefrontal and basal ganglia circuit alterations revealed by functional magnetic resonance imaging particularly implicating reward substrate (147–152). These changes may predispose patients to further dysexecutive or cognitive dysfunction important for progression to ICDs (146). Patients with pathological gambling show reduced frontal lobe activity during the Iowa Gambling Task (153). These patients also exhibit dysfunction of the mesocorticolimbic network (i.e., abnormal activity and blood flow in a network including the orbitofrontal cortex, cingulate cortex, hippocampus, amygdala, insula, and ventral pallidum) (154, 155). In one PD patient with hypersexuality, single-photon emission computed tomography (SPECT) imaging revealed increased medial temporal blood flow (156). Functional magnetic resonance imaging studies show increased ventral striatal activation in dopamine-medicated PD patients with pathological gambling and buying exhibited during rewarding outcomes (150). Imaging studies also demonstrate that with acute dopaminergic therapy, dopamine release in the ventral striatum is abnormal in patients with ICDs compared to non-ICD patients during reward wanting (68, 150, 157). Patients most susceptible to ICDs appear to have relatively preserved limbic-paralimbic neural architecture, suggesting a predisposition to dopaminergic overdosing of the reward system (158). With continued efforts, imaging will continue to define network-level alterations to potentially assist with the assessment, diagnosis, and treatment of ICDs in PD.

Aside from imaging, a vast literature has characterized the electrophysiology of the basal ganglia during action control and reward processing, both highly relevant processes for impulse control (108). Few studies have examined the electrophysiology of PD patients with ICDs. PD patients with ICDs have proportionally more reward-responsive neurons and less loss-responsive neurons in the STN (159). In a stop signal task with a small sample of 10 PD patients, STN high frequency (35–75 Hz) oscillatory activity decreased during inhibition (160). However, in the four patients with ICDs included in the study, this observation was not seen. It is unclear what the physiological meaning of this high frequency activity is, but it demonstrates the possibility of measuring meaningful electrophysiological pathology in the basal ganglia. In a separate study, relative to PD patients without ICD, PD patients with ICDs exhibited stronger differences in low frequency (2–12 Hz) power between risky and non-risky gambling decisions (161). Lastly, a study of nine PD patients with ICDs and without dopamine-induced dyskinesias found more STN theta (4–7.5 Hz) activity that was associated with similar theta activity in the premotor and frontal cortex (162). This signal may reflect the prominent role of the STN as a hub of response inhibition in the basal ganglia, perhaps through the hyperdirect pathway with the neocortex, which has been implicated in impulsivity (106). Together with imaging work, electrophysiological characterization of impulsivity will continue to remain as a valuable endeavor for pathophysiological insight and for motivating innovative neuromodulatory treatment modalities.

### Cognitive and Neuropsychological Factors

Studies of cognition in PD patients with ICDs have been extensively reviewed elsewhere (163) but generally implicate brain regions found to be dysfunctional through imaging studies. Importantly, PD patients with dementia exhibit lower rates of ICDs, suggesting that they likely do not exhibit global cognitive impairment (14). However, studies have also associated low MMSE or MOCA scores to ICDs even after controlling for numerous other variables such as age at onset and motor severity (38, 73). Other studies have examined targeted cognitive domains, such as the Iowa Gambling Task, in which PD patients with ICDs show poor decision making compared to age, sex, education, and disease severity matched PD controls (17). Across the four main subtypes, ICD patients have impaired spatial planning and set shifting (63). Patients with hypersexuality in particular are selectively impaired on the Stroop test, a behavioral paradigm testing attention and inhibition (63). Another study found Stroop deficits in a PD ICD cohort relative to non-ICD PD patients but did not include ICD subtype analyses (164). However, not all studies are in agreement about Stroop deficits (17). With the exception of PD individuals with pathological gambling, PD patients with ICDs have lower performance on verbal learning and memory tasks (63). PD patients with pathological gambling and shopping show faster gain learning during a probabilistic reward task (150). These differences across subtypes may reflect abnormal cortical regions specific to certain ICD subtypes. For instance, given these neuropsychological profiles, hypersexuality may implicate the temporal and frontal lobes, whereas pathological gambling may be more frontal-specific.

## Conclusion

In this review we have provided an overview of the numerous associations and risk factors for ICD-onset in individuals with PD. The review reveals that these factors vary considerably across samples and cultures, however some of the most consistent associations include dopaminergic medications, male gender, young age, early PD onset, longer disease duration, smoking, and increased impulsivity or novelty seeking personality traits. These characteristics may raise flags for clinicians as they consider patients at risk for impulsivity. Other risk factors discussed above, such as deep brain stimulation and non-dopaminergic medication use, have been less consistently established and will require further studies before definitive conclusions can be drawn. Although we have chosen to focus on the most common associations, there are several others that were not discussed here but may gain more research attention in the coming years, including socioeconomic status (21, 46), education (8, 74), and marriage status (8).

It is important to consider the many limitations in the studies presented in this review. In the overwhelming majority of cases, the studies are retrospective, observational, and utilize small sample sizes, although several large studies do exist (8, 28, 43). Across the various methodologies utilized, there are considerable differences in data collection. Numerous screening tools exist and may influence selection bias due to false positive or false negative ICD cases. For instance, compared to the modified Minnesota Impulsive Disorders Interview, the Questionnaire for Impulsive Compulsive Disorders in Parkinson's Disease Rating may overestimate ICD rates (5, 142), although some head to head comparisons have revealed similar rates (35). Some studies rely on private screening whereas others use self-administered assessments (5, 165). Another major limitation may be the time scale of a study. Although many studies consider cumulative incidence, cross-sectional prevalence and its connection to certain risk factors is difficult to accurately assess. For example, there can be a substantial time lag between dopamine agonist use and ICD onset (166). In addition, inclusion and exclusion criteria differ across studies and therefore results must not be hastily generalized to populations until external validity has been clearly established.

Remarkably, whether PD confers additional risk for ICD remains debated. Despite the strong associations between ICDs and PD characteristics like impulsivity traits, male gender, and increased depression, some studies conclude that PD patients in general are not at a particularly higher risk of ICDs (25, 142). A dearth of studies have compared unmedicated PD patients to non-PD controls and found no difference in ICD prevalence (142), however unmedicated PD patients differ greatly from those with more advanced disease. Another solution is to study other samples of non-PD patients that are treated with dopaminergic agents, although this does not account for cases of ICDs in PD that are unrelated to dopamine treatment (167). Nonetheless, it is useful to study ICDs in PD-specific cohorts with hopes of tailoring treatment strategies specific to this complex disease.

In conclusion, with few exceptions the literature surrounding ICDs in PD is vastly mixed and further research is greatly needed in many areas. We believe the literature presently supports that PD patients are uniquely susceptible to ICDs through numerous potential risk factors discussed in this review. For instance, one profound example of susceptibility with respect to impulsive behaviors comes from a hallmark animal experiment in which preference for alcohol after an STN lesion depended critically on preference for alcohol prior to surgery (100). There exists a complex relationship between susceptibility and impulsivity outcomes, and parallels may be drawn to DBS where after surgery patients can experience improvement, worsening, or no change in preoperative impulsivities. It is necessary to appreciate that analyses at the group level can mask this type of important individual variability. In addition to the numerous environmental and non-environmental risks discussed throughout this review, ICDs are likely related to susceptibility factors involving specific cognitive dysfunctions or neural circuitries (63, 150). Additionally, susceptibilities may differ across the heterogeneity of ICD subtypes. Specific ICDs can result from intrinsic reward hypersensitivities (e.g., sexuality) or learned ones (e.g., gambling) (146) dependent on cultural factors, genetics, and neuropsychiatric profiles (21, 46, 59, 128, 146). Clinicians should bear in mind the potential influences of prior history, current behaviors, and treatment modalities as they may relate to ICD behaviors in PD patients.

## Author Contributions

RE designed and wrote the paper with contributions from AR-Z, SC, and BP. RE and ZP-C designed the figures and ZP-C created the figures. All authors provided critical input and edits.

### Conflict of Interest Statement

The authors declare that the research was conducted in the absence of any commercial or financial relationships that could be construed as a potential conflict of interest.
